# Characterizing Sleep Disturbance Subgroups and Identifying Associated Factors in Traditional Chinese Medicine Nurses: A Latent Profile Analysis and Explainable Machine Learning Approach

**DOI:** 10.1155/jonm/1269507

**Published:** 2026-02-24

**Authors:** Chong Liu, Nieran Lian, Kristin K. Sznajder, Cong Li, Changqing Zou, Xiaoshi Yang

**Affiliations:** ^1^ Department of General Surgery, Shengjing Hospital of China Medical University, Shenyang, Liaoning, China, cmu.edu.cn; ^2^ College of Health Management, China Medical University, Shenyang, Liaoning, China, cmu.edu.tw; ^3^ College of Medicine, Pennsylvania State University, Hershey, Pennsylvania, USA, psu.edu; ^4^ School of Medical Humanities, China Medical University, Shenyang, Liaoning, China, cmu.edu.tw

**Keywords:** explainable machine learning, female nurses, latent profile analysis, sleep disturbance

## Abstract

**Background:**

Nurses in traditional Chinese medicine (TCM) departments face significant sleep challenges associated with occupational stressors. However, person‐centered analyses classifying these sleep patterns remain scarce. This study aimed to identify heterogeneous sleep disturbance subgroups via latent profile analysis (LPA) and evaluate the performance of explainable machine learning models in discriminating these subgroups based on demographic and occupational features.

**Methods:**

A cross‐sectional survey enrolled 7721 nurses from 130 TCM healthcare institutions in Liaoning Province (December 2024). Data encompassed demographic, occupational, and psychological variables obtained via self‐administered questionnaires, including the Patient‐Reported Outcomes Measurement Information System (PROMIS) Sleep Disturbance short form 8a. LPA was employed to categorize sleep disturbance patterns. Recursive feature elimination with random forest (RFE‐RF) was used to select features associated with subgroup membership for five machine learning models. Models were trained on 70% of the data and evaluated on a 30% independent test set. The optimal classification model (XGBoost) underwent interpretability analysis using Shapley additive explanations (SHAP).

**Results:**

LPA identified three subgroups: mild‐stable (29.8%), moderate‐fluctuating (60%), and severe‐persistent (10.2%). Machine learning models achieved test AUCs of 0.71–0.84, with XGBoost demonstrating the highest discriminatory performance (AUC = 0.84, 95%CI: 0.83–0.85) in classifying subgroups. SHAP analysis indicated that monthly income, organizational support, hospital level, self‐compassion, and resilience were the top five features contributing to the model’s classification output.

**Conclusion:**

This study characterized three distinct sleep disturbance subgroups among TCM nurses, with the majority exhibiting moderate symptoms. The sequential application of LPA and explainable machine learning demonstrated robust performance in distinguishing sleep disturbance patterns. Identifying correlates—such as lower income and resilience—may assist nurse managers in stratifying risk and tailoring interventions for those most likely to fall into the severe subgroup. Future longitudinal studies are required to validate the stability of these subgroups and establish causal relationships.

## 1. Introduction

Sleep disorders represent a critical global public health challenge characterized by abnormalities in sleep quality, duration, and rhythm, concurrent with significant daytime functional impairments. These disorders are frequently associated with cognitive decline, a higher prevalence of anxiety/depression, and profound socioeconomic implications related to reduced care quality and productivity [1]. Epidemiological studies indicate that the prevalence of sleep disorders in the general Asian population ranges between 26.4% and 39.4% [2]. Notably, nurses face an acute sleep health crisis, with Japanese cross‐sectional data showing insomnia prevalence (29.2%) 3–4 times higher than the general population [3]. This phenomenon is strongly associated with occupational specialties—shift‐induced circadian disruption, high workload, and chronic workplace stress collectively characterize systemic sleep threats [4, 5]. Female nurses demonstrate 1.3–2.0‐fold higher odds of poor sleep quality, a disparity correlated with the complex interplay among hormonal fluctuations, emotional labor, and social role pressures [6, 7].

Within TCM nursing, female nurses exhibit unique multidimensional profiles of sleep dysfunction. As practitioners bridging TCM and Western modalities, their occupational stressors manifest dual characteristics: (1) TCM‐specific interventions (e.g., acupuncture, herbal therapy, and emotional regulation) are linked to higher cognitive load, which correlates with prolonged sleep latency [8] and (2) health education mandates under the “preventive treatment” paradigm are associated with nonlinear role burden accumulation. Conflicts between traditional values (e.g., professional ethics glorifying overwork) and modern efficiency are further correlated with an increased prevalence of sleep disturbances in TCM nurses [9], highlighting this population’s theoretical and practical research significance.

Heilemann’s adaptation [10] of Lee’s conceptual framework [11] posits that chronic insomnia relates to interactions among three domains: personal factors (intrinsic traits/socioeconomic status), sleep deprivation factors (parental/professional roles, marital status, and living arrangements), and sleep disruptive factors (physiological/psychological/environmental triggers). Empirical studies identify 16 factors associated with nurses’ sleep quality spanning demographics (age and marital status), occupational factors (hospital level and night shifts), psychological states (anxiety and resilience), and organizational support [12–14]. These factors provide foundational evidence for identifying potential targets for support.

Currently, many studies on nurses’ sleep quality rely on total scores from sleep‐related scales for assessment, while paying less attention to the heterogeneity within the nurse population [15, 16]. Heterogeneity reflects differences in individual characteristics, behaviors, and needs, implying that different nurses may face distinct sleep‐related issues [17]. Traditional variable‐centered analytical approaches fail to capture this diversity. To address this, person‐centered strategies like latent profile analysis (LPA) have been increasingly adopted. For instance, Han et al. [18] classified nurses into high‐ and low‐symptom groups based on sleep scores, while Slavish et al. [19] characterized three sleep profiles—“poor overall sleep,” “nightmares only,” and “good overall sleep”—using sleep diaries. However, such LPA‐based studies often lack interpretability in defining subgroup boundaries—a limitation that parallels challenges in clinical screening and triage. However, while effective for classification, such LPA‐based studies often lack interpretability regarding the determinants of subgroup boundaries—a limitation that hinders the translation of latent phenotypes into clinical screening and triage tools.

Recent advances in explainable artificial intelligence (XAI) offer new avenues for interpreting complex clinical data. To address the heterogeneity of sleep disturbances, this study adopted a sequential analytical strategy combining LPA and XAI‐enabled machine learning [20–22]. Specifically, we aim to (1) objectively delineate latent sleep subgroups among female TCM nurses using LPA based on symptom characteristics; (2) subsequently employ machine learning models to discriminate subgroup membership using high‐dimensional demographic and occupational factors; and (3) apply SHAP to visualize the contribution of these features to the model’s classification output. This approach bridges the gap between unsupervised phenotype discovery and clinical application, allowing for the identification of key correlates to support stratified interventions.

## 2. Methods

### 2.1. Study Design and Participants

The sample size was calculated using the formula for estimating a single proportion: $n=\left(Z_{12-\alpha /}^{2}P\left(1-P\right)\right)/\varepsilon^{2}$. Based on a 95% confidence interval (*Z* = 1.96), a margin of error (*ε*) of 2%, and an estimated sleep disturbance prevalence (*P*) of 31.6% derived from prior research using the PROMIS scale [23], the initial sample size was determined to be 2076. To account for an anticipated nonresponse rate of 15%, the final required sample size was adjusted to 2443.

A cross‐sectional study employing multistage cluster sampling was conducted between December 2 and 9, 2024. In this study, 7721 nurses were recruited from 130 TCM hospitals and general hospitals in Liaoning Province. Stratified sampling based on geographical regions was performed, with 26 hospitals selected from each administrative division (east/west/south/north/central), culminating in cluster sampling. The inclusion criteria comprised individuals who met the following conditions: (1) possession of a registered nurse license, (2) aged 18 or above, (3) female gender, and (4) employment as a frontline nurse in TCM departments. Before questionnaire administration, participants provided informed consent. Exclusion criteria encompassed (1) nurses on leave at the time of the survey and (2) nurses diagnosed with or experiencing severe psychiatric disorders like bipolar disorder, schizoaffective disorder, and paranoid psychosis.

### 2.2. Data Collection

Survey data were collected via Wenjuanxing (https://www.wjx.cn), a widely used online survey platform in China. The Nursing Department of each hospital distributed survey links through institutional WeChat groups to ensure representativeness, enabling comprehensive access to all eligible nurses. Participation was voluntary and strictly anonymous. Mandatory response settings were implemented for all items to minimize missing data, with IP address restrictions preventing duplicate submissions. Rigorous quality control measures excluded 801 invalid responses (e.g., duplicates, patterned responses, or implausible entries), yielding a final analytic sample of 6920 valid responses (response rate: 89.63%).

### 2.3. Outcome and Measures

#### 2.3.1. The Sleep Disturbance Assessment

Sleep disturbance was assessed using the PROMIS Sleep Disturbance short form 8a [24], which evaluates sleep quality, depth, and restoration over the past 7 days. The scale consists of 8 items rated on a 5‐point Likert scale (1–5), and following the PROMIS scoring manual, selected items were reverse‐scored before raw scores were summed and converted to standardized T‐scores (mean = 50, SD = 10) [25]. In this metric, higher T‐scores reflect more severe sleep disturbance symptoms, thereby enabling the characterization of individuals with clinically significant sleep‐related symptoms within this population.

### 2.4. Features

Features potentially associated with sleep disturbances were categorized into three domains: individual factors, sleep deprivation factors, and sleep disruption factors. Individual factors encompassed age (≤ 45 vs. > 45 years), monthly income (< 3000 vs. ≥ 3000 RMB), educational attainment (high school or below vs. college or above), professional title (junior vs. senior/intermediate), organizational support (assessed via the perceived organizational support questionnaire [26]), psychological resilience (measured by the ego resilience scale [27]), and self‐compassion (evaluated using the self‐compassion scale‐short form [28]). Sleep deprivation factors included marital status (married vs. others), hospital level (tertiary vs. nontertiary), weekly working hours (≤ 40 vs. > 40 h), and monthly night shifts. Sleep disruption factors comprised chronic comorbidities, anxiety severity (assessed via the 7‐item generalized anxiety disorder scale (GAD‐7) [29]), depressive symptoms (measured by the 9‐item patient health questionnaire (PHQ‐9) [30]), stress perception (evaluated using the 10‐item perceived stress scale (PSS‐10) [31]), and fatigue levels (quantified through the 14‐item fatigue scale (FS‐14) [32]).

### 2.5. Statistical Analysis

This study excluded cases with missing data from the final analyses to prevent potential bias. Given the large sample size, these exclusions did not significantly compromise statistical power. The analytic sample (*N* = 6920) was first used to identify underlying sleep disturbance subgroups. LPA was employed for this purpose, which revealed three distinct profiles: mild‐stable (29.8%), moderate‐fluctuating (60.0%), and severe‐persistent (10.2%). Descriptive analyses of the complete sample were performed accordingly. Categorical variables were presented as counts (percentages) and analyzed using chi‐square or Fisher’s exact tests. Continuous variables were assessed for normality using the Kolmogorov–Smirnov test, with non‐normally distributed variables reported as median (*Q*_1_, *Q*_3_).

Subsequently, to develop a machine learning model for classifying these LPA‐derived subgroups, the dataset was randomly partitioned into a 70% training set and a 30% independent test set. The severe class imbalance observed in the full sample was preserved within the training set. To mitigate classifier bias toward the majority classes, the synthetic minority over‐sampling technique for nominal and continuous features (SMOTE‐NC) was applied exclusively to the training set. The test set remained completely separate and untouched to ensure an unbiased evaluation of model generalizability. Next, the discriminative contribution of features was evaluated on the resampled training data, followed by feature preprocessing (including Min–Max normalization).

Feature selection was conducted using recursive feature elimination with cross‐validation (RFECV) to identify the optimal feature subset. A random forest classifier served as the base estimator, initialized with all 16 candidate features (random seed fixed for reproducibility). The procedure iteratively pruned the least significant feature (step = 1) based on Gini importance rankings, with performance evaluated via 10‐fold stratified cross‐validation. The analysis demonstrated that the highest mean cross‐validation accuracy was achieved using the complete feature set; consequently, all 16 features were retained for final model construction. Subsequently, Min–Max normalization was applied to the feature matrix.

The training set was used for all model development, including hyperparameter tuning, while the test set was reserved exclusively for the final, unbiased evaluation of classification performance. All analyses were conducted using SPSS 26.0 and Python 3.7.11.

### 2.6. LAP

PROMIS Sleep Disturbance short form 8a served as indicators for identifying sleep disturbance subgroups using robust maximum likelihood estimation. Multiple statistical criteria were employed to determine the optimal number of latent profiles: entropy, Lo‐Mendell‐Rubin adjusted likelihood ratio test (LMR), bootstrap likelihood ratio test (BLRT), Akaike information criterion (AIC), Bayesian information criterion (BIC), and adjusted Bayesian information criterion (aBIC) [33–35]. Entropy values (range: 0–1) quantified classification accuracy, with higher values indicating better separation between latent classes. Following methodological recommendations [33], we adopted an entropy threshold > 0.80 to ensure precise profile assignment and minimize between‐group overlap, which is critical for reliable model interpretation. The LMR and BLRT statistics compared k‐class versus (*k* − 1)‐class models, where significant *p*‐values favored the more complex k‐class solution. Lower AIC, BIC, and aBIC values indicated superior model fit by balancing complexity against goodness‐of‐fiction [36]. The final determination of the profile number incorporated these fit statistics and theoretical considerations.

### 2.7. ML Models Development

Five machine learning algorithms—support vector machine (SVM), categorical boosting (CatBoost), multilayer perceptron (MLP), random forest (RF), and XGBoost—were employed to construct sleep disturbance subgroup classification models. A 5‐fold cross‐validation strategy was used for model training and internal validation. Hyperparameter tuning was performed for each algorithm through grid search with cross‐validation to optimize classification accuracy. The optimal hyperparameter configuration was selected based on cross‐validation accuracy and the area under the receiver operating characteristic curve (AUC‐ROC) in the training set. Model performance was evaluated using multiple indicators: ROC curve analysis (AUC), precision, specificity, and F1‐score (Table 1). Classification performance was assessed via 5‐fold cross‐validation to ensure robustness and mitigate overfitting. Discrimination ability was quantified through ROC analysis and AUC values, while calibration plots evaluated the agreement between classified probabilities and actual subgroup assignments. Decision curve analysis (DCA) was used to estimate the clinical utility and net benefit of the classification approach. Feature importance and feature attribution for subgroup classification were quantified using SHAP, where higher absolute values indicated a greater contribution to the model’s classification output. Additionally, we characterized feature value distributions and their statistical correlations with model outputs to elucidate model behavior further. Figure S1 (Supporting Information) displays the entire workflow of this study.

**Table 1:** Evaluation of five algorithm performances.

Algorithm	Data set	Precision	Recall	Specificity	F1	AUC
CatBoost	Train	0.719	0.7188	0.8594	0.716	0.8782
	Test	0.6081	0.6124	0.8062	0.607	0.7856

SVM	Train	0.5632	0.5648	0.7824	0.5558	0.7581
	Test	0.5273	0.5294	0.7647	0.5189	0.7163

MLP	Train	0.6709	0.6753	0.8376	0.6698	0.8478
	Test	0.5614	0.5682	0.7841	0.5606	0.7564

Random forest	Train	0.5874	0.5855	0.7927	0.5792	0.7609
	Test	0.5332	0.5342	0.7671	0.5263	0.7119

XGBoost	Train	0.9155	0.9149	0.9575	0.9145	0.9844
	Test	0.658	0.664	0.832	0.6588	0.8368

*Note:* AUC, area under the curve; CatBoost, categorical boosting; MLP, multilayer perceptron; XGBoost, extreme gradient boosting.

Abbreviations: RF, random forest; SVM, support vector machine.

### 2.8. Ethical Considerations

This study received ethical approval from the Research Ethics Committee of China Medical University (approval No. 2020048). Before their inclusion in the study, all participants provided written informed consent.

## 3. Results

### 3.1. LAP of Sleep Disorders Among TCM Nurses

**Figure 1:**
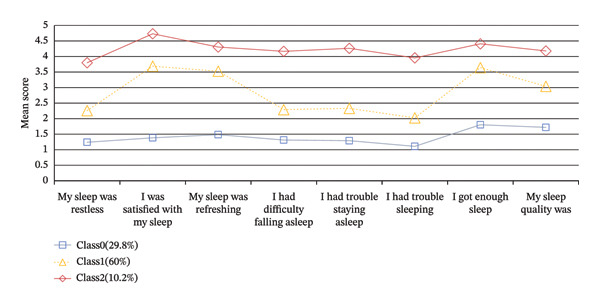
Latent profile analysis of sleep disturbances in female nurses.

As shown in Table 2, as the number of latent classes increased from 1 to 5, the AIC, BIC, and aBIC values demonstrated a decreasing trend, reflecting the optimization process between model complexity and data fit. All classification solutions exhibited excellent class separation, with entropy values consistently above 0.9. Both BLRT and LMR tests achieved statistical significance across all models. The three‐class model was selected as the optimal solution, balancing statistical fit with interpretability and parsimony. Compared to the two‐class model, the three‐class solution showed a substantial improvement in fit and yielded a clear, theoretically coherent characterization of sleep disturbance patterns (see Figure 1). Although models with four and five classes had slightly lower information criteria, the improvement was marginal and did not offer more distinct clinical profiles. The three‐class model also demonstrated excellent classification accuracy, with an entropy value of 0.946. This finding holds significant implications to inform targeted nursing management strategies.

**Table 2:** The model fit indices for latent profile analysis of sleep disorders among female nurses.

Profile	AIC	BIC	aBIC	Entropy	LMR	BLRT	Proportion
1	177581.210	177690.685	177639.841	—	—	—	—
2	156995.021	157166.075	157086.631	0.939	*p* < 0.001	*p* < 0.001	0.32327/0.67673
3	147224.552	147457.186	147349.142	0.946	*p* < 0.001	*p* < 0.001	0.29783/0.60014/0.10202
4	140100.823	140395.037	140258.393	0.905	*p* < 0.001	*p* < 0.001	0.30303/0.35029/0.25708/0.08960
5	137039.558	137395.351	137230.107	0.923	*p* < 0.001	*p* < 0.001	0.08165/0.28974/0.25578/0.29783/0.07500

Figure 1 displays the score distributions across items for each latent class. Based on the scoring patterns, Class 0 (29.8%) was designated as the “mild‐stable sleep disturbance group,” Class 1 (60.0%) as the “moderate‐fluctuation sleep disturbance group,” and Class 2 (10.2%) as the “severe‐persistent sleep disturbance group.”

### 3.2. Descriptive Statistics

The study enrolled 6920 female nursing professionals. LPA categorized sleep disturbances into three severity subgroups: mild‐stable (*n* = 2061), moderate‐fluctuation (*n* = 4153), and severe‐persistent (*n* = 706) sleep disturbances. To address the class imbalance within the training partition, SMOTE‐NC was applied to exclusively the training partition. This approach generated synthetic samples based on nearest neighbors to augment the minority classes without contaminating the hold‐out test set, yielding a balanced dataset of 12,459 samples. Within this expanded dataset, 5408 nurses (43.41%) were characterized by lower sleep disturbance levels (*T* < 50), while 7051 nurses (56.59%) demonstrated higher levels of sleep disturbance (*T* ≥ 50). Baseline characteristics for the training and testing sets are presented in Table 3. The distributions of demographic, occupational, and psychological variables were highly similar between the two sets (all *p* > 0.05), indicating a successful random partitioning of samples. This supports the comparability of the sets for model training and validation while mitigating potential bias arising from data partitioning.

**Table 3:** Baseline characteristics between training and testing sets.

Variables	Total (*n* = 12459)	Training set (*n* = 8721)	Testing set (*n* = 3738)	Statistic	*p*
Age, *n* (%)				0.74	0.388
≤ 45	11616 (93.23)	8142 (93.36)	3474 (92.94)		
> 45	843 (6.77)	579 (6.64)	264 (7.06)		
Marital status, *n* (%)				1.61	0.204
Married	9949 (79.85)	6938 (79.56)	3011 (80.55)		
Others	2510 (20.15)	1783 (20.44)	727 (19.45)		
Education level, *n* (%)				0.04	0.845
Junior college or below	4132 (33.16)	2897 (33.22)	1235 (33.04)		
University or above	8327 (66.84)	5824 (66.78)	2503 (66.96)		
Professional title, *n* (%)				2.51	0.113
Junior	7090 (56.91)	5003 (57.37)	2087 (55.83)		
Senior/intermediate	5369 (43.09)	3718 (42.63)	1651 (44.17)		
Monthly income, *n* (%)				1.15	0.284
< 3000	5985 (48.04)	4162 (47.72)	1823 (48.77)		
≥ 3000	6474 (51.96)	4559 (52.28)	1915 (51.23)		
Weekly working hours, *n* (%)				0.01	0.932
≤ 40	3793 (30.44)	2653 (30.42)	1140 (30.50)		
> 40	8666 (69.56)	6068 (69.58)	2598 (69.50)		
Chronic diseases, *n* (%)				0.02	0.889
Yes	973 (7.81)	683 (7.83)	290 (7.76)		
No	11486 (92.19)	8038 (92.17)	3448 (92.24)		
Hospital level, *n* (%)				1.71	0.192
Tertiary	4386 (35.20)	3102 (35.57)	1284 (34.35)		
Nontertiary	8073 (64.80)	5619 (64.43)	2454 (65.65)		
Night shifts, *n* (%)				0.00	0.961
Yes	6287 (50.46)	4402 (50.48)	1885 (50.43)		
No	6172 (49.54)	4319 (49.52)	1853 (49.57)		
Anxiety, *M* (*Q*_1_, *Q*_3_)	0.14 (0.00, 0.33)	0.14 (0.00, 0.33)	0.14 (0.00, 0.33)	−0.77	0.442
Depression, *M* (*Q*_1_, *Q*_3_)	0.11 (0.00, 0.30)	0.11 (0.00, 0.30)	0.07 (0.00, 0.30)	−1.30	0.193
Organizational support, *M* (*Q*_1_, *Q*_3_)	0.61 (0.50, 0.81)	0.63 (0.50, 0.81)	0.61 (0.50, 0.80)	−1.27	0.205
Resilience, *M* (*Q*_1_, *Q*_3_)	0.62 (0.43, 0.74)	0.62 (0.43, 0.74)	0.62 (0.43, 0.74)	−0.30	0.764
Perceived stress, *M* (*Q*_1_, *Q*_3_)	0.36 (0.30, 0.36)	0.36 (0.30, 0.36)	0.36 (0.30, 0.36)	−0.76	0.445
Self‐compassion, *M* (*Q*_1_, *Q*_3_)	0.54 (0.50, 0.67)	0.54 (0.50, 0.67)	0.54 (0.50, 0.67)	−0.52	0.605
Fatigue, *M* (*Q*_1_, *Q*_3_)	0.58 (0.25, 0.83)	0.58 (0.25, 0.83)	0.58 (0.25, 0.83)	−0.37	0.714

*Note: M*, median; *Q*_1_, first quartile (indicating 25% of data values fall below this point); *Q*_3_, third quartile (denoting 75% of data values lie below this point).

### 3.3. Feature Selection for Sleep Disorders Among TCM Nurses

**Figure 2:**
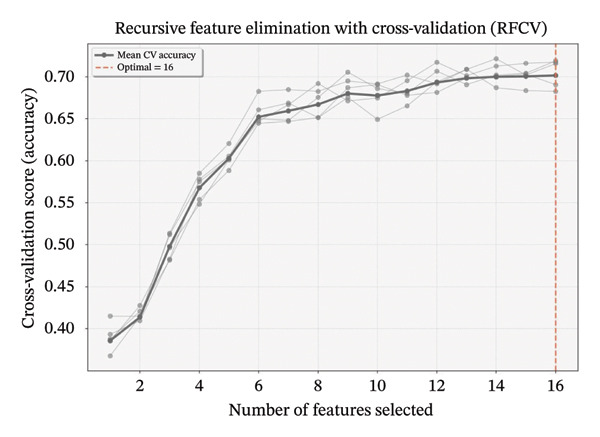
Feature selection using recursive feature elimination with cross‐validation. Note: the recursive feature elimination based on random forest (RF‐RFE) analysis demonstrates feature selection performance across validation iterations. Gray lines depict accuracy scores for varying feature quantities in each of the 5 cross‐validation folds, while the bold black line represents mean cross‐validation accuracy. The optimal feature count (16) is indicated by the red dashed line. Recursive Feature Elimination with Cross‐Validation (RFCV) denotes the same method as the RFECV described in the text.

To identify variables most strongly associated with sleep disturbance profiles, we performed recursive feature elimination with cross‐validation (RFECV) using a random forest estimator. Starting with an initial pool of 16 variables, the algorithm recursively eliminated features with the lowest importance weights. This yielded an optimal feature subset comprising 16 variables. In Figure 2, gray lines represent accuracy scores for different feature quantities across five cross‐validation iterations; the bold black line indicates mean cross‐validation accuracy, and the red dashed line identifies the optimal feature count (16). The final classification model incorporated age, marital status, education level, professional title, monthly income, weekly working hours, chronic diseases, hospital level, night shifts, anxiety, depression, organizational support, resilience, perceived stress, self‐compassion, and fatigue symptoms.

### 3.4. Comparative Analysis of Multiple Classification Models

Following feature selection, five algorithms—SVM, CatBoost, MLP, RF, and XGBoost—were evaluated using 5‐fold cross‐validation and grid search. XGBoost emerged as the optimal architecture. While the model exhibited high discriminative capacity in the training phase, the performance on the independent held‐out test set remained robust (AUC 0.84; 95% CI: 0.83–0.85) (Figure 3). Given the class imbalance, DCA was conducted to assess clinical utility beyond standard metrics. The DCA results (Supporting Figure S2) confirmed that the XGBoost model offers a consistently positive net benefit across a broad range of preference thresholds, substantiating its reliability for classifying the severe‐symptom subgroup. Consequently, XGBoost was selected as the foundational classifier for the subsequent SHAP interpretability analysis (hyperparameters in Table S1).

Figure 3ROC curves of the training set and test set. Note: comparative analysis of receiver operating characteristic (ROC) curves for five machine learning models across training and test datasets. Train set panels: (a) Class 0 ROC curve; (b) Class 1 ROC curve; (c) Class 2 ROC curve; and (d) macro‐averaged ROC curve. Test set panels: (e) Class 0 ROC curve; (f) Class 1 ROC curve; (g) Class 2 ROC curve; and (h) macro‐averaged ROC curve.

**Figure:**
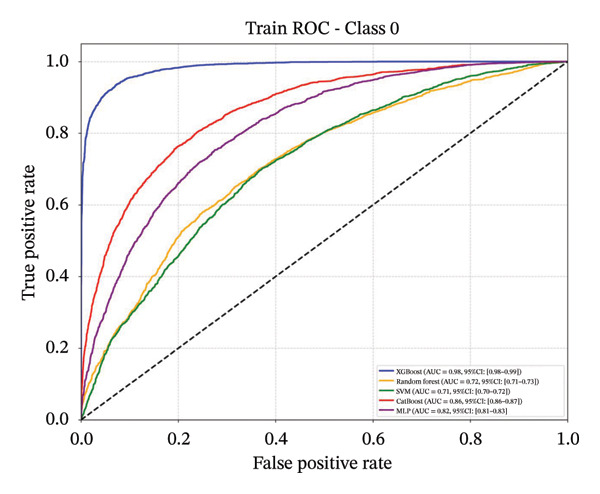
(a)

**Figure:**
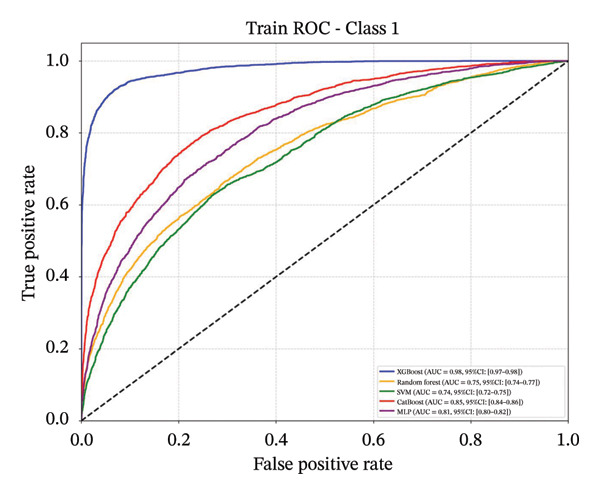
(b)

**Figure:**
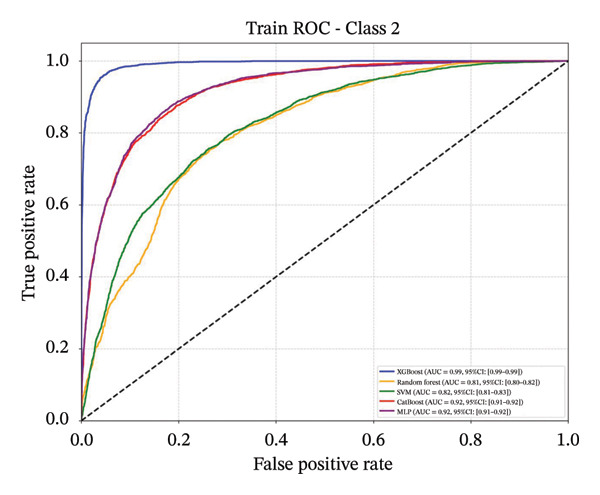
(c)

**Figure:**
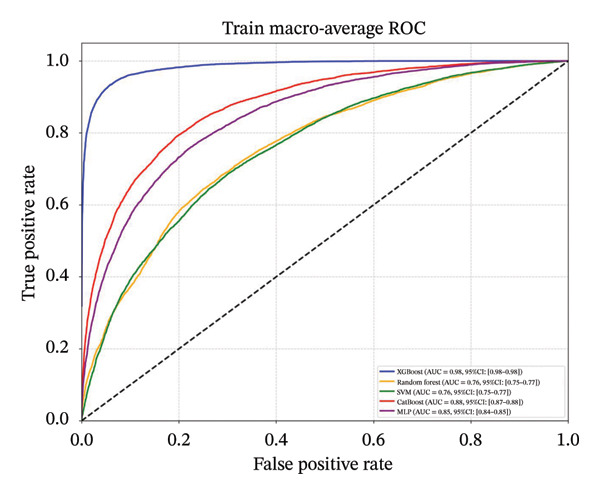
(d)

**Figure:**
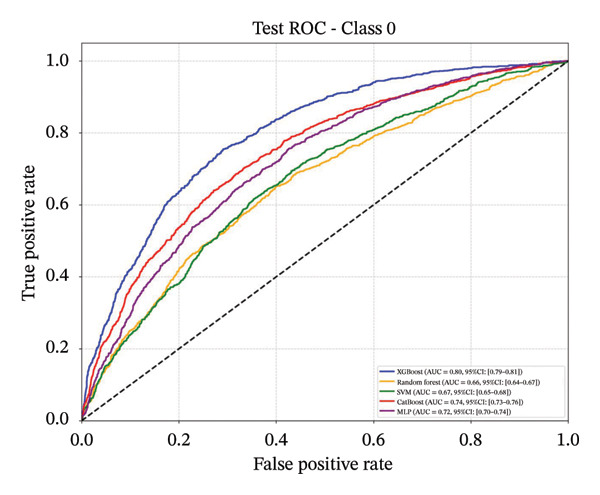
(e)

**Figure:**
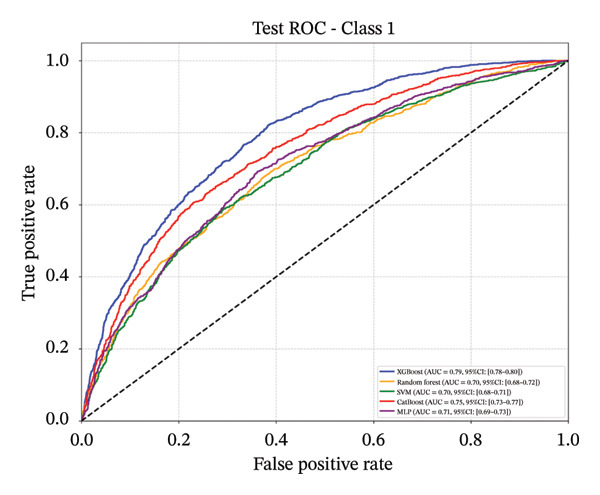
(f)

**Figure:**
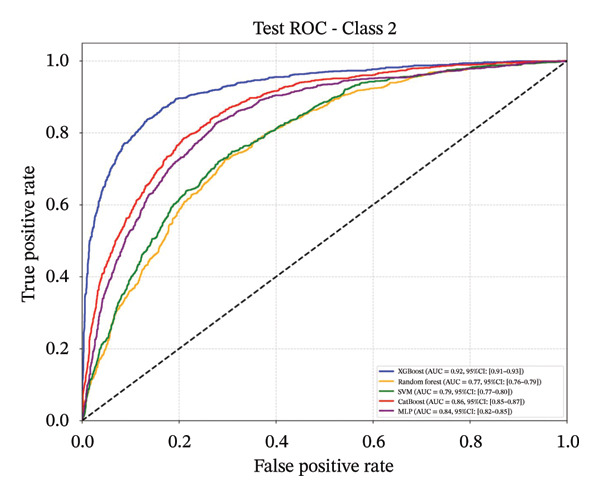
(g)

**Figure:**
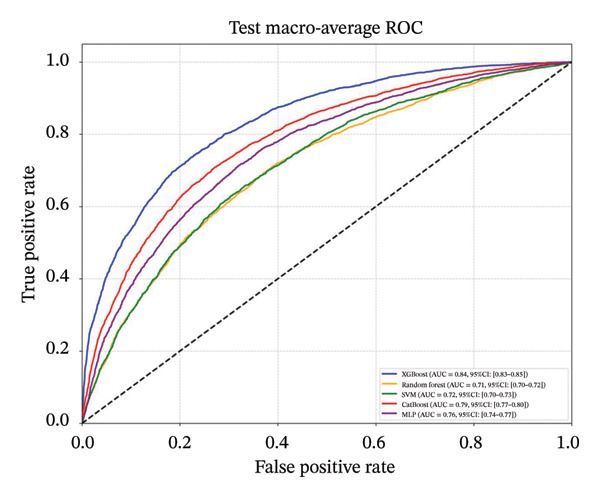
(h)

### 3.5. Model Interpretation

Figure 4 presents the interpretability analysis using SHAP values, which quantify each feature’s contribution to the model’s classification output (Figure 4(a)). Specifically, Figures 4(b), 4(c), and 4(d) display the importance rankings of the 16 features for each sleep disturbance subgroup. Conceptually, a SHAP value indicates the magnitude and direction of a feature’s association with deviations from the average estimated probability (base value). Vertical ordering reflects relative discriminative importance within the model. Force plots (Figures 4(e), 4(f), and 4(g)) decompose individual classifications by visualizing the cumulative additive contributions of features, while dependence plots (Supporting Figure S3) further capture nonlinear associations between feature values and model output. Collectively, this framework elucidates the mathematical logic driving the classifier within the current dataset. It is crucial to note that these feature contributions reflect cross‐sectional statistical associations identified by the algorithm, rather than confirmatory longitudinal causality.

Figure 4SHAP analysis results of XGBoost model. Note: SHAP (Shapley additive explanations) analysis visualization. (a) Mean‐based variable importance ranking across subgroups. (b) Summary plot for Class 0; (c) summary plot for Class 1; (d) summary plot for Class 2. Each panel displays all samples and features by category, with rows representing individual features and the *x*‐axis indicating SHAP values. Red dots denote higher feature values, while blue dots indicate lower values. (e) SHAP force plot for Class 0 samples; (f) SHAP force plot for Class 1 samples; (g) SHAP force plot for Class 2 samples. Red arrows represent elevated risk of disability, with blue arrows indicating reduced risk. Arrow length corresponds to effect magnitude, where longer arrows denote more significant predictive contributions.

**Figure:**
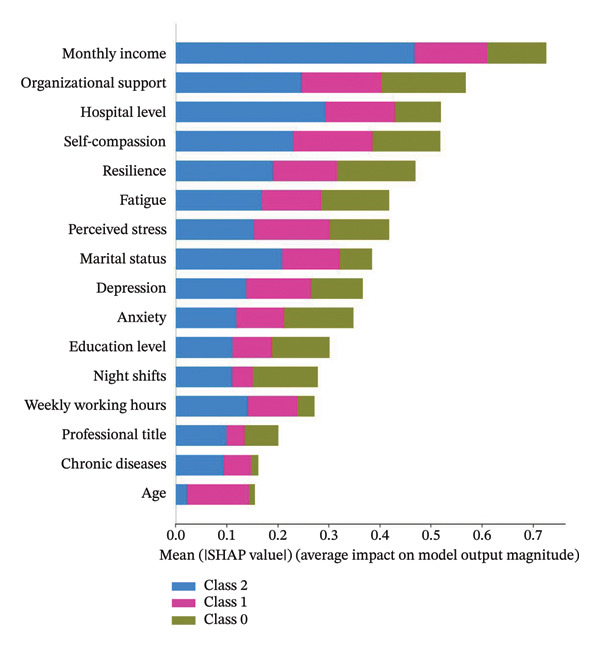
(a)

**Figure:**
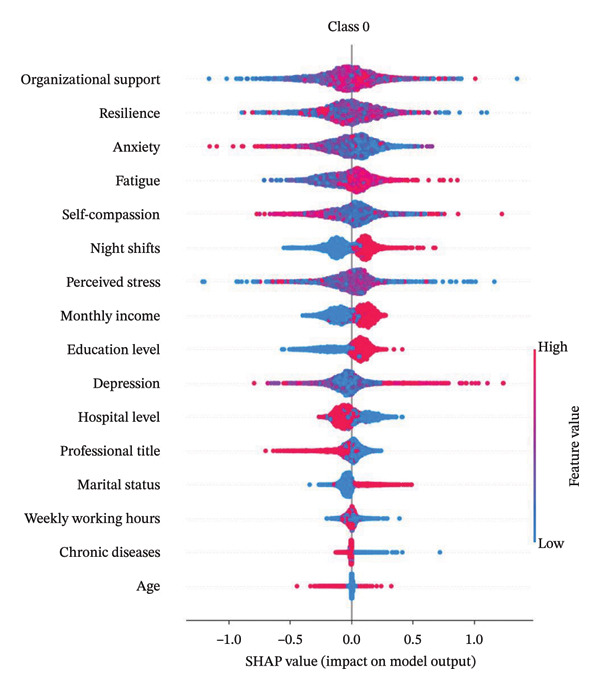
(b)

**Figure:**
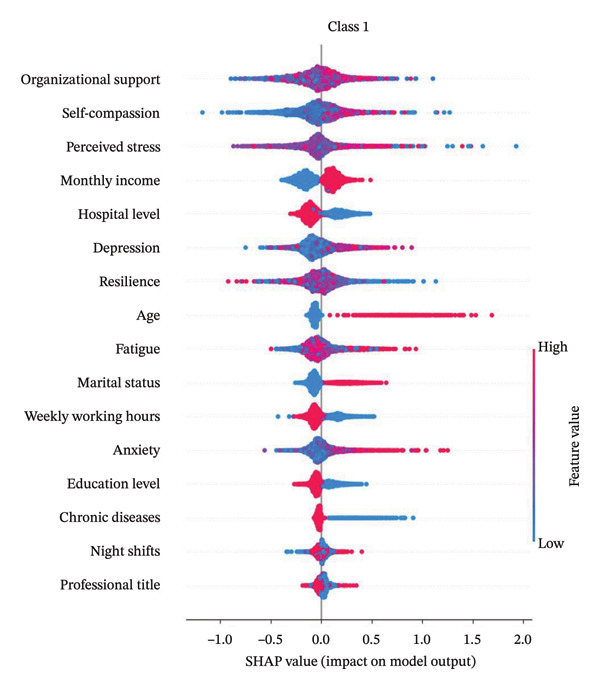
(c)

**Figure:**
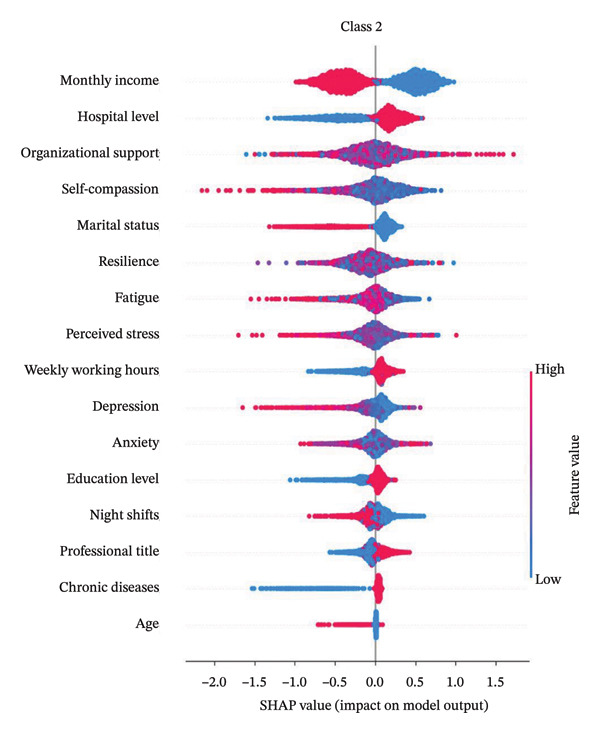
(d)

**Figure:**
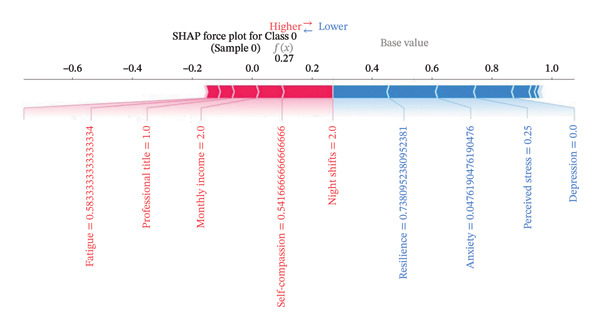
(e)

**Figure:**
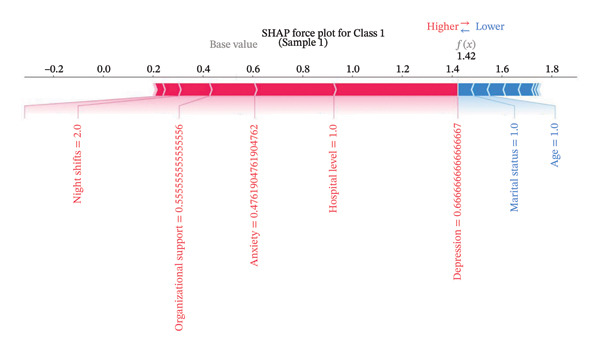
(f)

**Figure:**
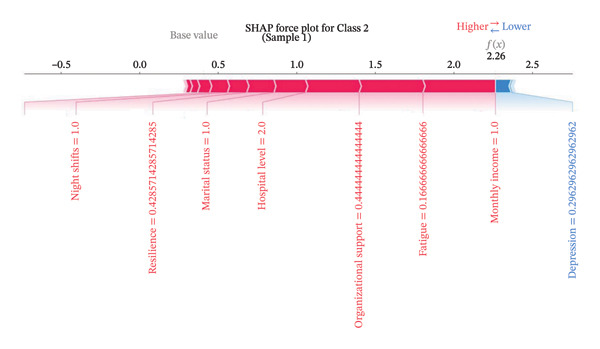
(g)

## 4. Discussion

### 4.1. LAP of Sleep Disturbance Among Female Nurses in TCM Departments

Among the participants, 5408 nurses (43.41%) were characterized by lower sleep disturbance levels (*T* < 50), while 7051 nurses (56.59%) demonstrated higher levels of sleep disturbance (*T* ≥ 50). These findings indicate a relatively high prevalence of sleep disturbance symptoms among nurses in TCM departments in Liaoning Province, suggesting significant sleep‐related challenges that are associated with their occupational characteristics, psychological stress tolerance, and environmental adaptability.

LPA revealed three characteristic sleep disturbance profiles among female nurses in TCM departments: mild‐stable, moderate‐fluctuating, and severe‐persistent subgroups. These findings indicate significant interindividual heterogeneity in sleep disorders within this nursing population. This heterogeneity suggests the potential value of interventions aligned with the United Nations’ Sustainable Development Goals (SDGs), particularly SDG 3 (good health and well‐being) for sleep health promotion, SDG 5 (gender equality) addressing disproportionate caregiving burdens, and SDG 8 (decent work) targeting occupational stressors [37–39]. The subgroup‐specific patterns revealed here may provide evidence for precision public health strategies that advance these global agendas while addressing local workforce needs.

The mild‐stable sleep disturbance group (29.8% of participants) exhibited lower overall scores but higher ratings on “I got enough sleep” and “my sleep quality” items. This discrepancy suggests a divergence between subjective assessments and objective sleep quality, potentially associated with unrealistic beliefs (e.g., the “eight‐hour” myth). Addressing misconceptions and optimizing sleep hygiene might relate to a lower likelihood of progression toward chronic refractory sleep patterns. These targeted measures align with the United Nations’ SDGs through multiple pathways: sleep education programs directly advance SDG 3.4 (mental health promotion) and SDG 4.4 (life skills training), while workplace adjustments like flexible scheduling support SDG 8.5 (productive employment) by reducing work‐related sleep disruptions [37, 38, 40]. Such integrated interventions exemplify how health‐specific strategies can synergize with broader socioeconomic development targets.

The moderate‐fluctuating sleep disorder group had the highest number of people, accounting for 60% of the total participants, and scored relatively high on “I was satisfied with my sleep,” “I got enough sleep,” and “my sleep was refreshing”. These findings suggest that despite their positive perceptions of sleep, they may not obtain sufficient rest, leading to challenges in achieving restorative sleep. Night work disrupts the natural circadian rhythm, being associated with alterations in cortisol and melatonin production, disturbing the sleep–wake cycle, and hindering adaptation to diurnal and nocturnal activity patterns [41]. This circadian disruption can coincide with difficulties initiating sleep, shortened sleep duration, and reduced sleep quality. Research indicates that night‐shift nurses typically sleep less than 5 hours, contrasting with the average adult sleep duration of 7.2 h [42]. Strategies such as shortening night shifts, reducing consecutive night shifts, providing tranquil rest areas for night‐shift nurses, and aligning shift schedules with the body’s circadian rhythm are recommended to address these challenges. Implementing these circadian‐protective policies operationalizes SDG 8.8 (safe working environments) by mitigating shift‐related health risks, while real‐time fatigue monitoring systems leverage SDG 9.b (technology for well‐being) to prevent sleep deprivation [37, 43]. Notably, these interventions concurrently address SDG 5.4 (unpaid care work recognition) by reducing schedule‐induced conflicts between professional duties and domestic responsibilities—a critical gender equity consideration in this female‐dominated workforce [39].

The severe‐persistent group (10.2%) demonstrated uniformly elevated scores across all 8 PROMIS items, indicating clinically significant insomnia in this subset. Their balanced score profile suggests pervasive, nonsituational sleep disturbances requiring comprehensive intervention. The poor sleep quality of nurses is statistically linked to work‐related stress in addition to night shifts. Work stress among nurses primarily stems from heavy workloads, extended hours, strained doctor–patient relationships, and career advancement pressures. Research indicates a significant negative correlation between work stress and sleep quality, indicating that higher levels of work stress are associated with poorer sleep quality among nurses [44]. Moreover, when faced with high‐intensity work demands, nurses experience heightened psychological burdens, which co‐occur with anxiety, depression, and other emotional challenges, and are statistically associated with more severe sleep symptoms [45]. Therefore, it is recommended that hospitals may consider providing psychological support and enhance working conditions which might relate to a reduction in work‐related stress and improved sleep health. For this high‐risk subgroup, a multisectoral approach is needed to achieve SDG 3.4 (mental health promotion) through clinical insomnia treatment, while SDG 10.2 (social inclusion) mandates antistigma campaigns to overcome barriers to care‐seeking [38, 46]. Coordinating these efforts via SDG 17.17 (multistakeholder partnerships)—engaging hospitals, mental health services, and labor unions—can create sustainable support systems that address both individual sleep pathology and systemic occupational determinants [47].

### 4.2. Analysis of Associated Factors for Sleep Disorders Among Female Nurses in TCM Departments

In this cross‐sectional study, we employed a machine learning approach to identify factors associated with sleep disturbances and to classify nurses into distinct subgroups. Using the RF‐RFE method for feature selection, we derived a set of 16 salient variables. Five machine learning algorithms were then evaluated for their ability to construct a classification model based on these features. Among them, the XGBoost algorithm demonstrated the optimal discriminative performance in differentiating the predefined sleep disturbance subgroups. Interpretation of the optimal model via SHAP analysis identified features with the highest statistical weights for the classification, including monthly income, organizational support, hospital grade, self‐compassion, and resilience. Furthermore, the SHAP analysis quantified feature attribution, elucidating how each variable contributes to the model’s classification probability.

#### 4.2.1. Monthly Income

Monthly income is a significant factor associated with sleep disorders among nurses. In the mild‐stable and moderate‐fluctuating sleep disorder groups, lower income was associated with subgroup membership, consistent with Gao’s findings [48]. This reflects SDG 8.5’s call for equal pay and safe working environments, as financial stress correlates with increased emotional distress and poorer sleep quality. Financial constraints are often linked to residing in suboptimal environments (e.g., poor sound insulation), which co‐occurs with compromising sleep health, aligning with SDG 8.8’s principle of promoting safe working conditions [37]. Conversely, in the severe‐persistent group, higher income exhibited a negative association, aligning with Wang’s results [49]. This may stem from middle‐ to high‐income nurses demonstrating sleep patterns associated with higher professional responsibility and career advancement—a phenomenon highlighting the complex relationship between economic growth (SDG 8.1) and worker wellbeing. To address these correlates in line with SDG 8 targets, strategies such as housing subsidies or optimized scheduling might relate to reduced housing‐related stressors while promoting sustainable economic growth and productive employment [37].

#### 4.2.2. Organizational Support

This study revealed a dichotomous association between organizational support and sleep disturbance subgroups: positive correlations with mild‐stable and moderate‐fluctuating subtypes, but negative correlation with severe‐persistent patterns. This finding aligns with SDG 3’s emphasis on promoting mental health and well‐being, particularly for healthcare workers who face unique occupational stressors [38]. Given nursing’s female‐dominated nature, these findings also inform SDG 5’s gender equality targets, as organizational support systems correlate with better management of caregiving burdens [39]. Consistent with Zou’s findings [50], organizational support demonstrated a significant association with nurses’ sleep disorders. Strong perceived organizational support co‐occurs with higher levels of psychological capital and a lower prevalence of insomnia symptoms, while also potentially lower levels of gender‐specific stressors like workplace discrimination that disproportionately affect female nurses’ sleep quality [51]. Robust social support is associated with better sleep health, which statistically correlates with higher resilience [52] and lower negative emotionality [53]. Paradoxically, high support is also associated with higher odds of insomnia symptoms in some contexts [54], potentially explained by the frequent co‐occurrence of high support with high job demands, which are strongly associated with elevated stress. These complex relationships underscore the need for SDG 3‐aligned policies that holistically consider both organizational support systems and workload management in healthcare settings [38]. For affected nurses, strengthening organizational support is associated with lower levels of occupational stressors (e.g., reducing overcommitment) and boost psychological resources. Optimizing workflows (e.g., consolidating nocturnal care tasks) and implementing health feedback mechanisms would facilitate early sleep problem detection and systemic quality improvement.

#### 4.2.3. Hospital Level

Hospital level showed a negative association with mild‐stable and moderate‐fluctuating sleep disturbance subgroups but was positively associated with the severe‐persistent sleep disturbance group. Prior research indicates emergency nurses in tertiary hospitals exhibit the highest PSQI scores (11.8 ± 4.3), significantly exceeding those in secondary/primary hospitals [55], suggesting a positive correlation between hospital level and sleep problem severity. In tertiary hospitals, the complex caseloads and higher workloads are related with greater psychological stress, reduced autonomy, and weaker social support [56]. While their superior resources (e.g., structured training and career advancement) may be associated with lower stress in subthreshold‐to‐acute stages [57], the chronic experience of demanding environments (e.g., frequent night shifts and high‐intensity work) is linked to a higher prevalence of severe disturbance patterns [55]. These findings highlight the need for SDG 3‐aligned workplace interventions to protect nurses’ mental health, particularly in tertiary hospitals where sleep disturbance risks are highest. Implementing SDG 8‐compliant policies on reasonable working hours and rest periods could help mitigate these occupational hazards [37, 38]. Multilevel interventions targeting stressor management, support systems, and individual health promotion are advised for comprehensive sleep quality enhancement. Such measures would contribute to achieving both health‐related SDGs and decent work objectives in healthcare settings.

#### 4.2.4. Self‐Compassion

Self‐compassion showed positive associations with moderate‐fluctuating sleep disturbance but negative correlations with mild‐stable and severe‐persistent sleep disturbance subgroups. The association of self‐compassion with better sleep in the latter subgroups may be conceptually linked to (1) lower activity HPA axis and (2) enhanced activity of self‐soothing systems like oxytocin release [58–60]. These findings support SDG 3’s target of promoting mental health through psychological interventions [38]. However, excessive self‐focus in highly self‐compassionate individuals may paradoxically be associated with poorer sleep, potentially related to behaviors like bedtime procrastination [61]. Evidence‐based interventions such as mindfulness meditation, self‐talk exercises (e.g., “insomnia is transient”), and cognitive behavioral therapy for insomnia (CBT‐I) are designed to improve emotional regulation, correct maladaptive sleep habits (e.g., reducing time awake in bed), and reduce pre‐sleep negative cognition, which may relate to a reduction in procrastination behaviors. Such interventions align with SDG 4.4’s aim to enhance life skills for health promotion, while supporting SDG 8’s decent work principles through improved staff wellbeing [37, 40]. Healthcare institutions should integrate these approaches into workplace health programs to advance both individual and organizational sustainability goals.

#### 4.2.5. Resilience

This study demonstrates a significant negative correlation between nurses’ psychological resilience and sleep disturbances, consistently observed across various subgroups. Research confirms that nurses with higher resilience are associated with a lower prevalence of sleep disorders, as higher resilience is associated with better coping with work stress, occupational adversity, and traumatic events, which is linked to lower insomnia symptom severity [62]. Psychological resilience is associated with a weaker relationship between negative emotions and sleep disturbances and a reduced association of nightmares with poor sleep quality, potentially attenuating the statistical overlap within the anxiety‐depression‐insomnia cycle [63]. Notably, while night‐shift nurses face more severe circadian disruptions, highly resilient individuals tend to employ more effective coping strategies, which are correlated with lower insomnia‐related emotional distress [64]. These findings support SDG 3.4 (mental health promotion) and SDG 8.8 (safe working environments) [37, 38]. Factors that may explain the observed association between resilience and better sleep include stress reduction, emotional regulation, and the use of optimized coping strategies. Healthcare institutions should prioritize resilience training programs, aligning with SDG 4.4 (life skills development) while simultaneously advancing SDG 3 and 8 targets for staff wellbeing and working conditions [37, 38, 40].

### 4.3. Development of a Machine Learning Classification Model for Sleep Disorders Among Female Nurses in TCM Departments

This study developed the first machine learning model to identify and characterize sleep disturbance subgroups among female TCM nurses, aligning with multiple UN’s SDGs. Our SHAP‐derived key features transcend individual‐level characteristics by revealing systemic associative factors that bridge local sleep health management with the global SDG agenda. The model identifies modifiable factors—including resilience (SDG 3.4: mental health promotion), organizational support (SDG 3.8: health workforce capacity), and income (SDG 8.5: decent work)—that inform targeted interventions [37, 38]. For instance, resilience training (SDG 3.4) is hypothesized to help manage occupational stress‐induced insomnia, while gender‐inclusive policies (SDG 5.4) could mitigate caregiving‐work conflicts exacerbated by low support [38, 39].

Compared to traditional linear models, our XGBoost approach captures complex interactions among 16 variables with superior discriminative accuracy. Particularly, the statistical associations between low income and financial stress and poor organizational support to emotional exhaustion—both SDG 8.5/3.8 priorities—emerged as critical correlates of sleep disturbance. By identifying these factors, the model identifies potential targets for dual‐purpose interventions such as circadian‐aware shift reforms (SDG 8.8) and supportive income policies (SDG 8.5), which are associated with potential improvements in nurse wellbeing and advance institutional SDG compliance [37].

Implementation requires addressing China‐specific cultural barriers: underreporting due to mental health stigma (SDG 3.4 challenge) and gendered care expectations (SDG 5.4 gap) [38, 39]. By integrating objective metrics (work hours) with SDG‐aligned policies, healthcare systems can operationalize these findings while protecting privacy—a prerequisite for ethical SDG localization. Future validation should assess cross‐cultural generalizability to maximize global SDG contributions.

### 4.4. Limitations

This study has several limitations that should be acknowledged. Primarily, the cross‐sectional design precludes the establishment of causal inference among variables. Second, the exclusive inclusion of nurses from TCM departments in Liaoning Province may limit the generalizability of our findings. Third, the selected variables were constrained by the questionnaire structure, potentially omitting other relevant factors. The operationalization of variables (as categorical or continuous) and chosen cutoff points may also be associated with variations in findings. Finally, the use of PROMIS subjective measures might not fully align with participants’ actual sleep conditions. Future research should consider expanding the sampling framework, increasing sample size, and incorporating objective sleep monitoring devices to yield more robust evidence.

This study acknowledges several limitations. Primarily, the cross‐sectional design precludes establishing causal relationships among variables. Second, although a rigorous split‐sample strategy ensured internal validity, the reliance on a specific cohort of TCM nurses in Liaoning Province without external validation limits generalizability. Consequently, future multicenter studies across diverse regions and healthcare systems are essential to definitively establish the model’s robustness and transferability. Third, the scope of variables was constrained by the questionnaire structure, potentially omitting other relevant factors; moreover, the operationalization of variables (e.g., categorization and cutoff points) may introduce measurement bias and influence results. Fourth, reliance on self‐reported questionnaires raises concerns about potential biases and data quality, such as recall bias, social desirability bias, and common method bias. Specifically regarding sleep assessment, while the PROMIS Sleep Disturbance short form is validated, subjective reports may not fully align with objective physiological sleep parameters, potentially leading to misclassification or inaccurate estimation of sleep disturbance severity. The lack of objective sleep monitoring further limits the validation of the subjective sleep measures. Fifth, to mitigate the severe class imbalance in the training set, the SMOTE‐NC technique was employed. While applied exclusively to the training set to preserve the integrity of the test data, the reliance on synthetic data generation remains a methodological limitation that could theoretically influence the model’s decision boundaries. Therefore, future validation in larger, naturally balanced cohorts is recommended. Future research should employ longitudinal designs, expand sampling for broader generalizability, incorporate a wider range of variables and sensitivity analyses on operationalization, mitigate self‐report biases through study design, integrate objective sleep monitoring, and validate predictive models in naturally balanced samples to strengthen the evidence.

## 5. Conclusions

Adopting a person‐centered approach, we identified female nurses’ sleep quality into three subgroups: mild‐stable, moderate‐fluctuating, and severe‐persistent sleep disturbance clusters. Notably, 5408 nurses (43.41%) were characterized by lower levels of sleep disturbance symptoms (*T*‐score < 50), while 7051 (56.59%) demonstrated higher levels of sleep disturbance (*T*‐score ≥ 50). Machine learning analysis identified monthly income, organizational support, hospital level, self‐compassion, and resilience as key correlates associated with these distinct sleep patterns. These findings suggest that addressing these systemic and individual factors is essential for managing the severe‐persistent disturbance cluster. Strategies targeting these associated factors may offer a pathway to alleviate sleep disturbances in this population. Aligning such measures with evidence‐based solutions could support multiple SDGs by conceptually linking healthcare worker wellbeing (SDG 3), gender‐equitable workplaces (SDG 5), and decent working conditions (SDG 8) to sustainable healthcare systems.

## Author Contributions

Chong Liu designed the data collection protocol, contributed to the acquisition and analysis of data, and supervised the execution of the extensive multicenter survey across 130 participating hospitals. Nieran Lian performed the advanced statistical modeling (including LAP and machine learning algorithms), contributed to the analysis of data and revision of the manuscript, and drafted the original manuscript. Kristin K. Sznajder contributed to the English writing and linguistic refinement of the manuscript to ensure international publication standards and contributed to the revision of the manuscript. Cong Li and Changqing Zou mobilized regional resources and managed the logistical coordination of the data collection network to ensure ethical compliance. Changqing Zou contributed to the acquisition of data. Xiaoshi Yang conceived the overarching study design, secured funding, and provided supervision and final approval of the version to be published and was responsible for the conception, drafting, and revision of the manuscript. Chong Liu, Nieran Lian, and Kristin K. Sznajder are co‐authors.

## Funding

The authors received no specific funding for this work.

## Disclosure

All authors read and approved the final manuscript.

## Ethics Statement

This study was approved by the Ethics Committee of China Medical University (Ref no. 2020048) and conducted in line with Helsinki Declaration principles.

## Consent

All nurses participated voluntarily with consent after assurance of data privacy.

## Conflicts of Interest

The authors declare no conflicts of interest.

## Supporting Information

Figure S1: flow chart of the study.

Figure S2: calibration curves and decision curves for five machine learning models on the test set.

Figure S3: SHAP dependence plots for the XGBoost model.

Table S1: optimized hyperparameters for machine learning models.

## Supporting information


**Supporting Information** Additional supporting information can be found online in the Supporting Information section.

## Data Availability

The data that support the findings of this study are available on request from the corresponding author. The data are not publicly available due to privacy or ethical restrictions.
