# Supplementary material for: Characterizing Sleep Disturbance Subgroups and Identifying Associated Factors in Traditional Chinese Medicine Nurses: A Latent Profile Analysis and Explainable Machine Learning Approach
Source: J Nurs Manag. 2026 Feb 24;2026:1269507. doi: 10.1155/jonm/1269507 (PMC12931154; doi:10.1155/jonm/1269507)
Supplement: Supplementary file 1 — Supporting Information Additional supporting information can be found online in the Supporting Information section. [file JONM-2026-1269507-s001.docx]

**Predicting Sleep Disturbance Subgroups in Traditional Chinese Medicine Nurses: A Latent Profile Analysis and Explainable Machine Learning Approach**

**Appendix A1**

**Fig S1** Flow chart of the study


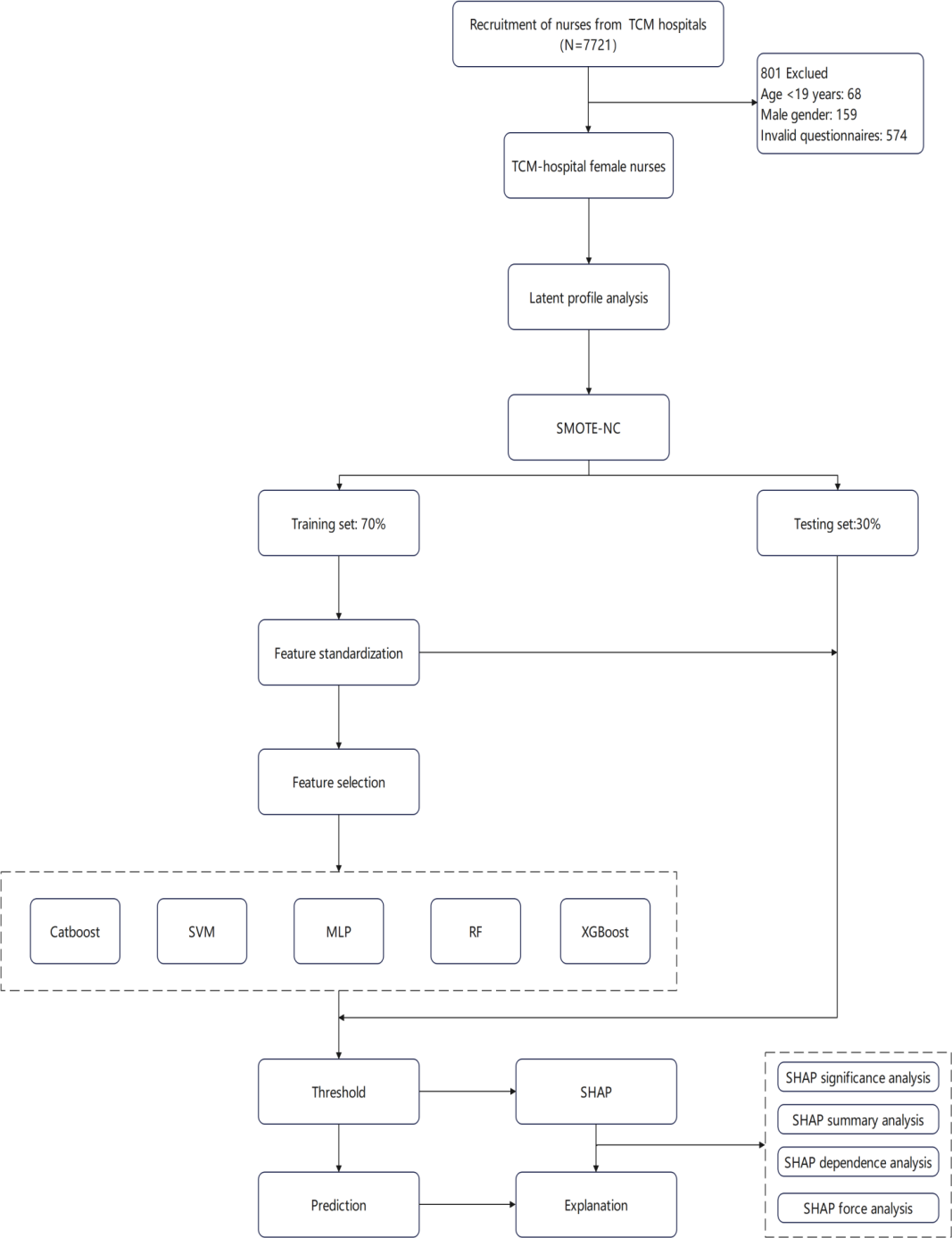


**Fig S2** Calibration curves and decision curves for five machine learning models on the test set.

(a)
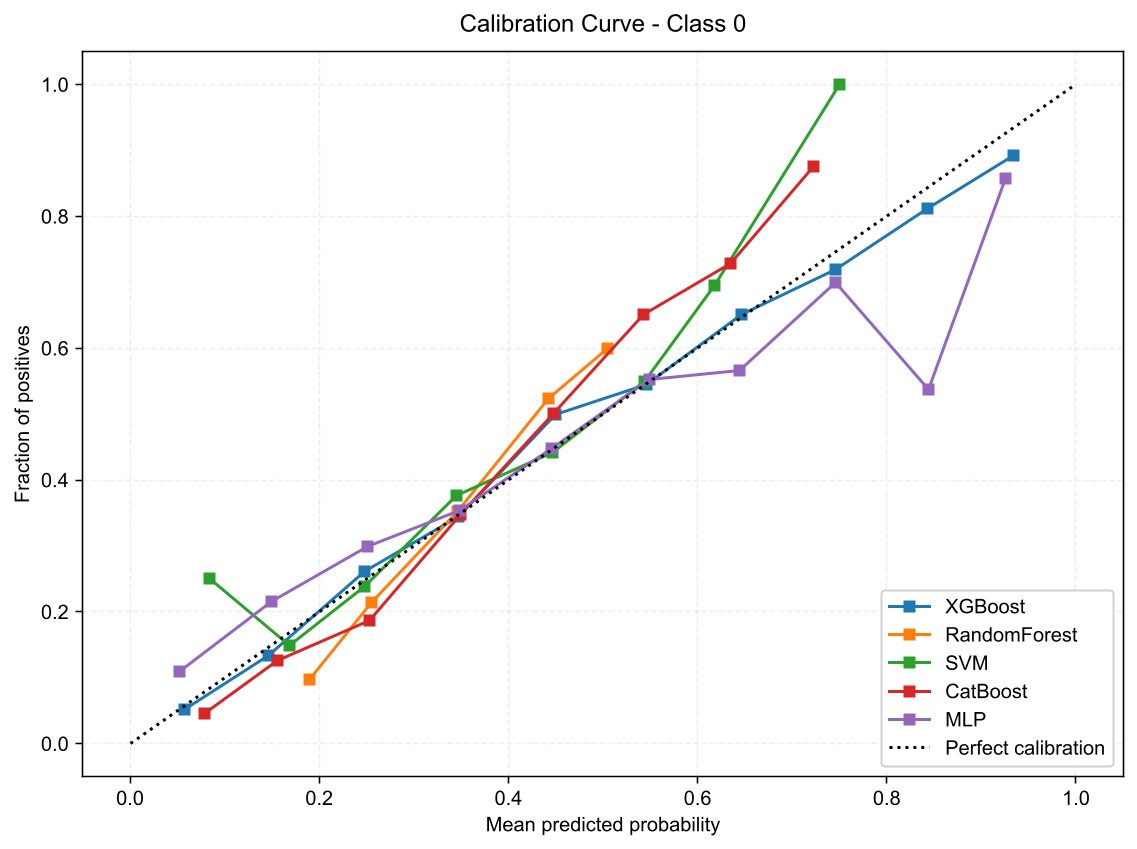


(b)
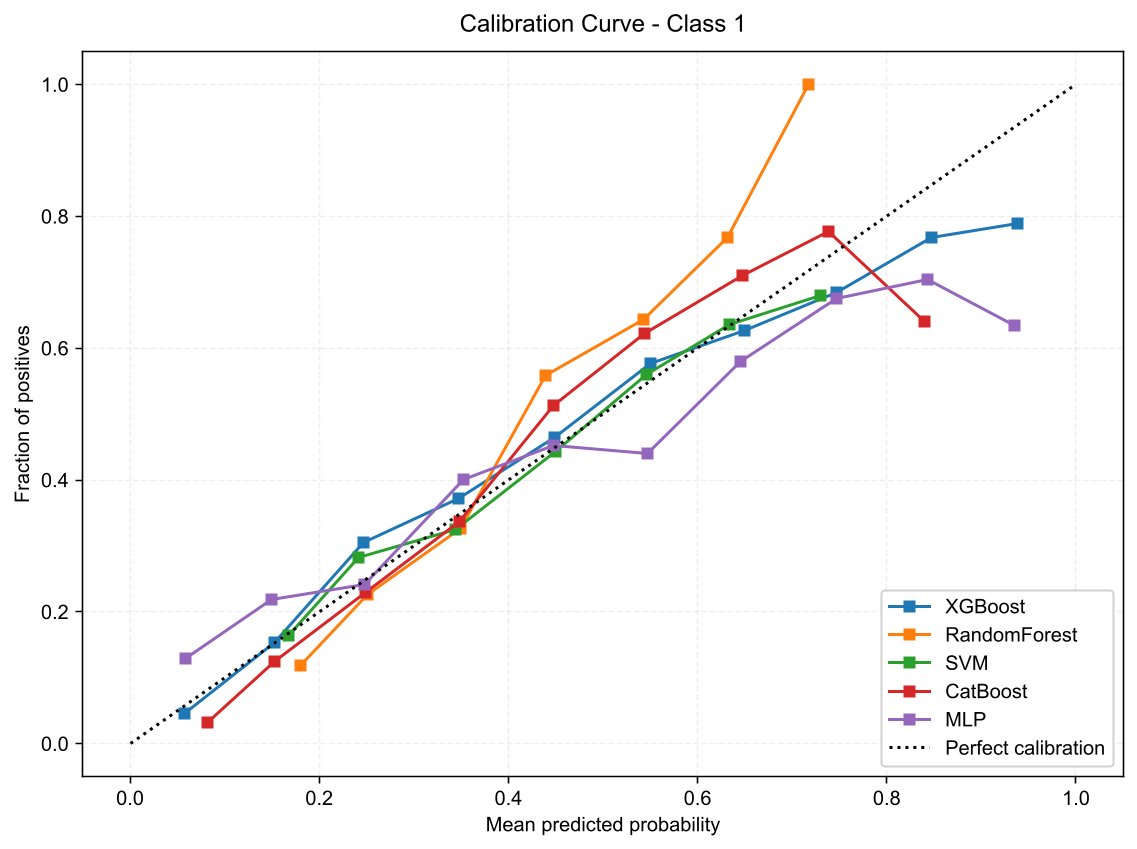


(c)
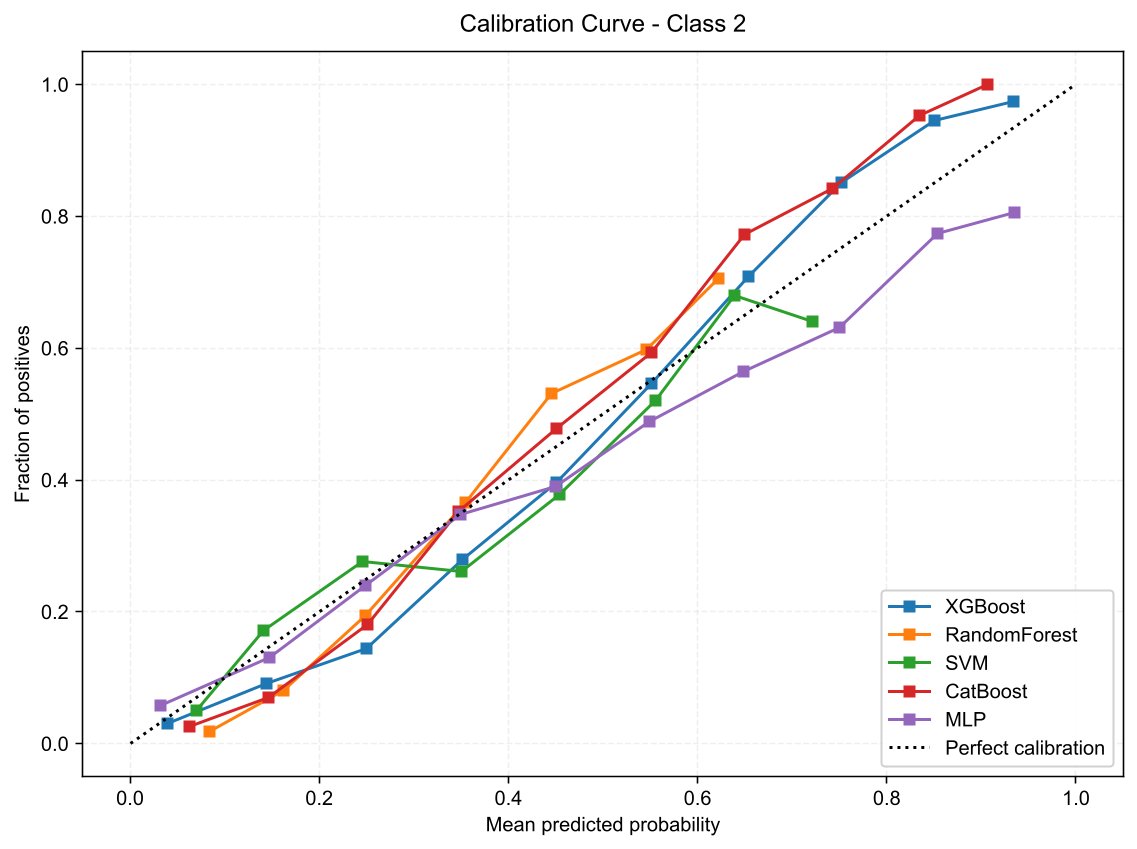


(d)
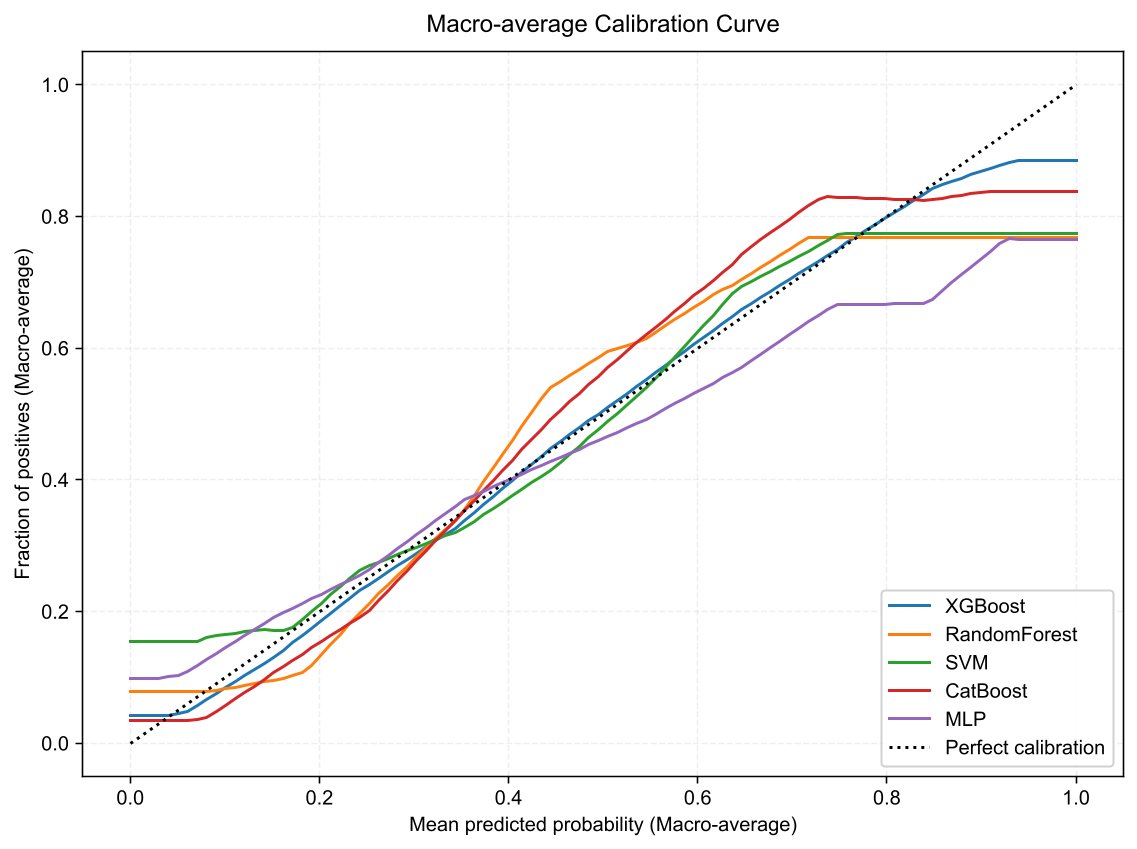


(e)
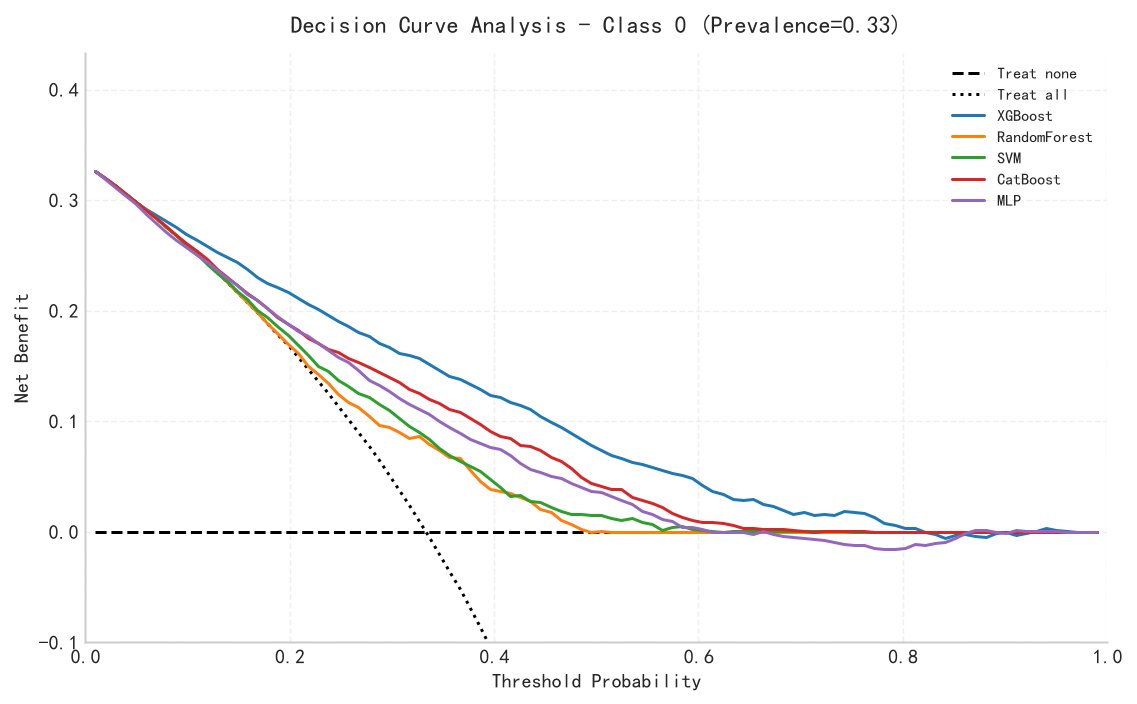


(f)
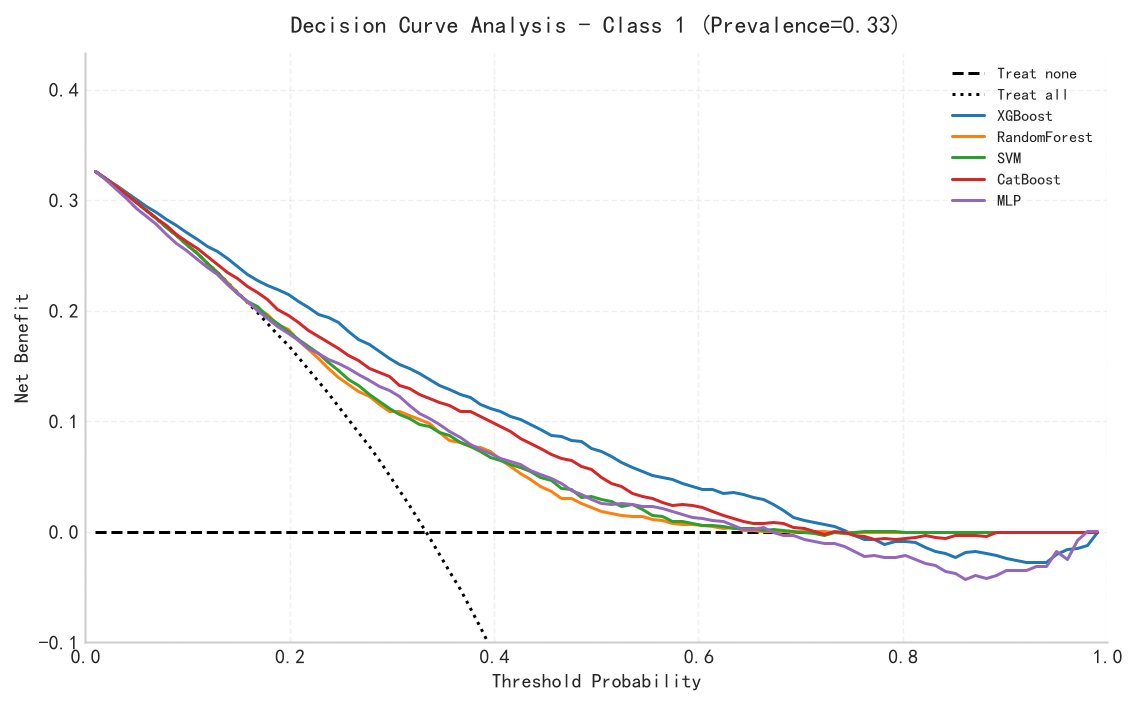


(g)
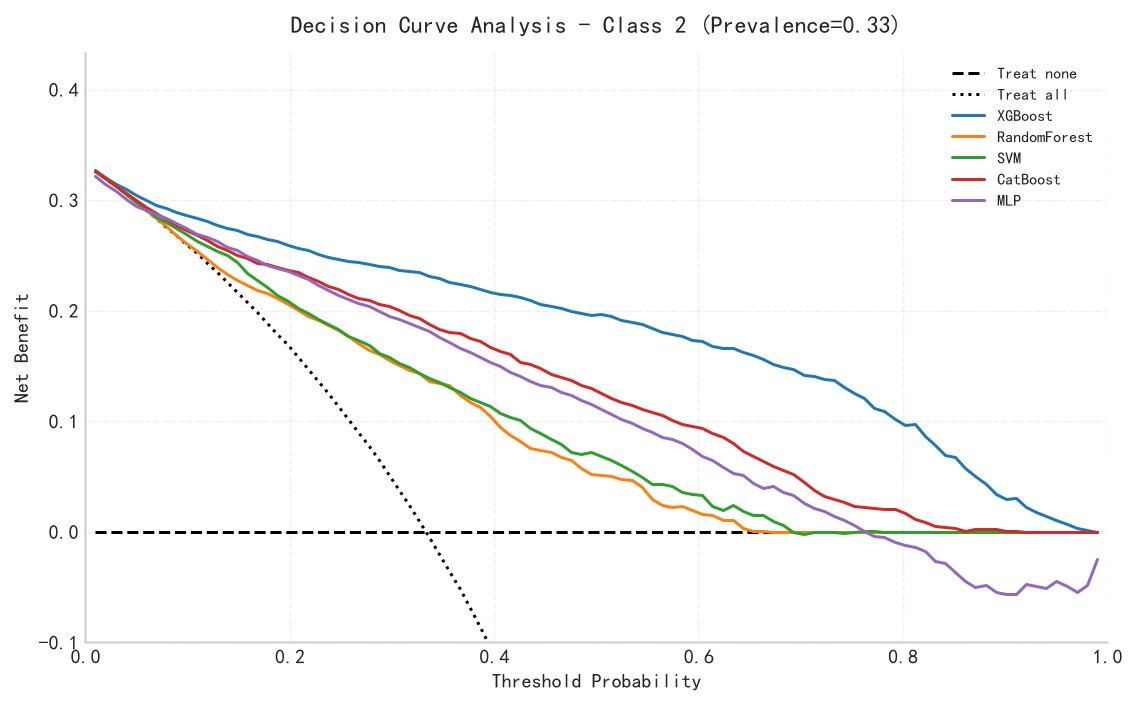


(h)
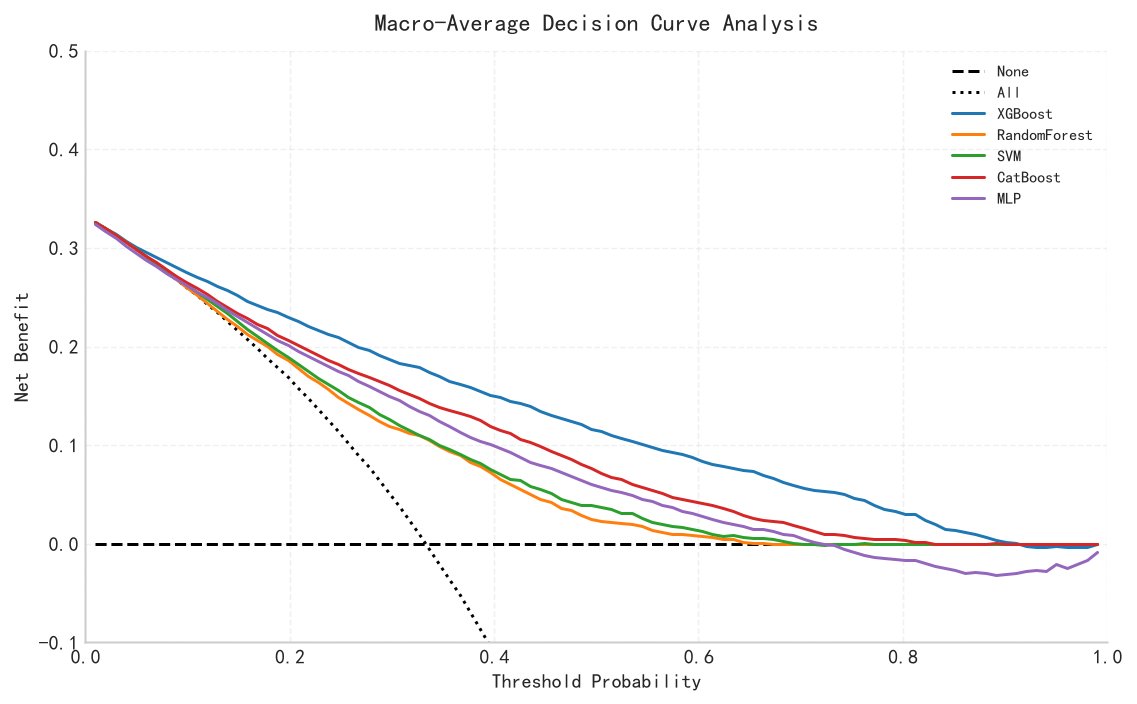


****Note****: Comparative evaluation of calibration curves and decision curves for five machine learning models on the test set. (a) Class 0 calibration curve; (b) Class 1 calibration curve; (c) Class 2 calibration curve; (d) Macro-averaged calibration curve; (e) Class 0 decision curve; (f) Class 1 decision curve; (g) Class 2 decision curve; (h) Macro-averaged decision curve.

**Fig S3** SHAP dependence plots for the XGBoost model.


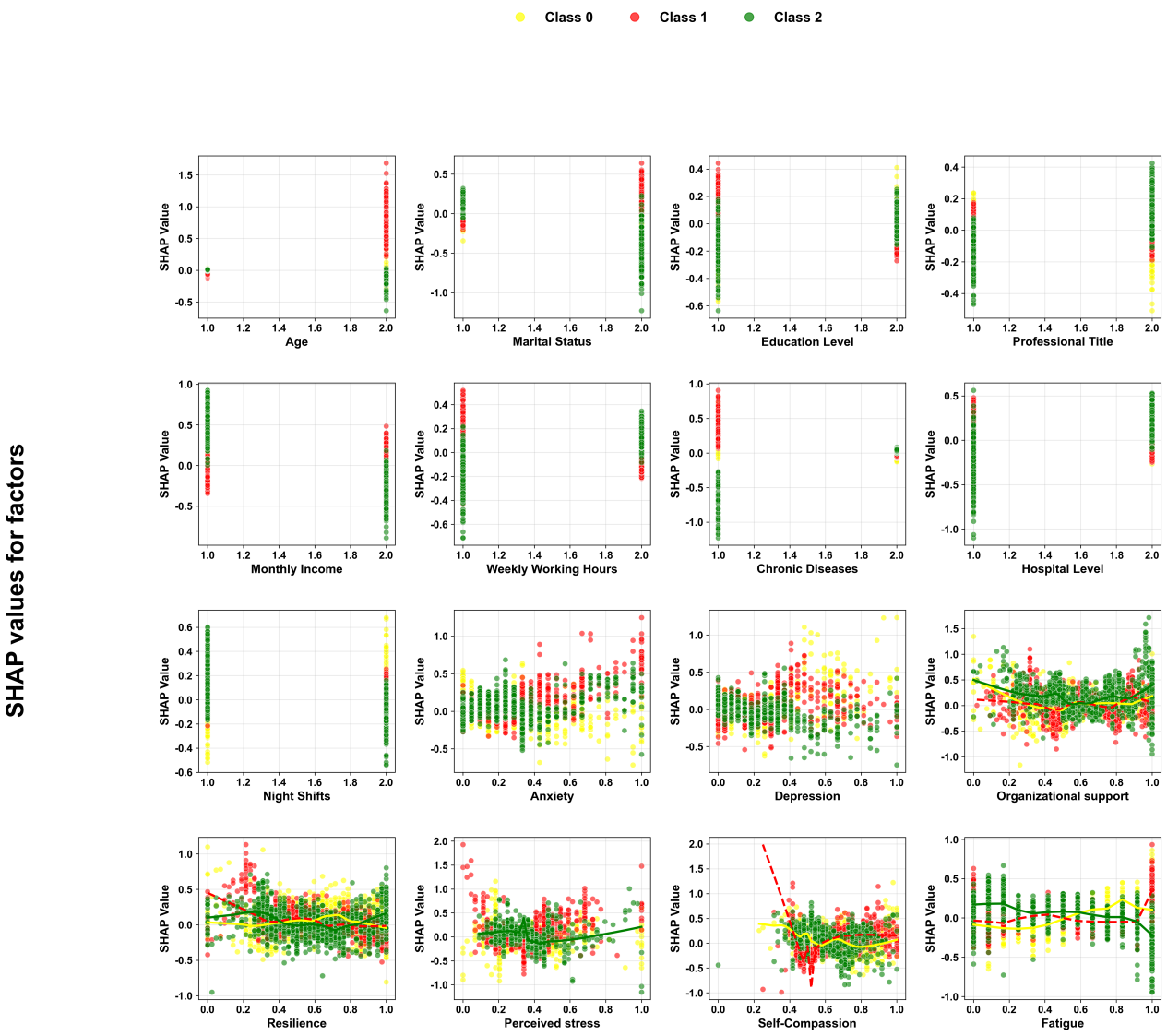


****Note****: SHAP dependence plots for the XGBoost model, with feature values on the x-axis and corresponding SHAP values on the y-axis. The scatter plot distribution reveals the feature's predictive influence across different value ranges. Color coding indicates sleep disturbance severity groups: yellow (mild), red (moderate), and green (severe).

**Table S1: Optimized Hyperparameters for Machine Learning Models**

| Model | Hyperparameter | Final Value |
| --- | --- | --- |
| XGBoost | learning_rate | 0.1 |
|  | max_depth | 6 |
|  | n_estimators | 300 |
| Random Forest | max_depth | 6 |
|  | n_estimators | 200 |
| SVM | C | 10 |
|  | kernel | 'rbf' |
| CatBoost | auto_class_weights | 'Balanced' |
|  | border_count | 64 |
|  | depth | 6 |
|  | iterations | 300 |
|  | l2_leaf_reg | 1 |
|  | learning_rate | 0.05 |
| MLP | alpha | 0.001 |
|  | hidden_layer_sizes | (50, 50) |

**Note:** CatBoost, categorical boosting; SVM, support vector machine; MLP, multilayer perceptron; RF, random forest; XGBoost, extreme gradient boosting.
